# Treating giant cell tumors: The eternal conundrum

**DOI:** 10.4103/0019-5413.32036

**Published:** 2007

**Authors:** Ajay Puri

**Affiliations:** Chairman - Indian Orthopedic Association (Oncology), Associate Professor - Orthopedic Oncology, Tata Memorial Hospital, Mumbai, India. E-mail: docpuri@vsnl.com

Bone tumors comprise less than 1% of all neoplasms and most orthopedic surgeons choose to refer them to specialist centers for management. Giant cell tumor of bone (GCT) is a possible exception. One of the commonest bone tumors encountered by an orthopedic surgeon, it is a lesion that continues to fascinate us. The routine orthopedic surgeon often fancies his chances against it and would endeavor to sally forth in his battle against it, albeit with varying results based on his experience and skills.

Usually benign, they are locally aggressive and may occasionally undergo malignant transformation. The first clinical collection of giant cell tumors was by Samuel Gross in 1879. In his paper “Sarcoma of long bones” he concluded that though they were the least aggressive of bone sarcomas they should not be regarded as innocent.^1^ The earliest reports of “curettage” were performed by Volkmann and reported by Krause in 1889. Joseph Bloodgood was the first to formulate a theory of management for “giant cell tumor” in 1912. He advocated the use of chemical adjuvants after curettage and performed bone grafting two to six weeks after removal of the gauze packed in at the time of curettage.^1^

Our understanding of giant cell tumor and strategies for management has evolved considerably over the last 100 years. The evolution of diagnostic and surgical techniques has helped considerably in early recognition and better delineation of these lesions thereby reducing recurrence rates compared to the historically reported recurrence rates of 50-60%. Though the dictates of function-preserving conservative surgery still hold good in management of giant cell tumors, the treating surgeon now has at his disposal a vast variety of reconstruction techniques if the need for segmental resection should arise. The availability of indigenous affordable prosthesis may enthuse many a young surgeon to advocate excision and replacement in large or recurrent GCTs. A megaprosthesis is an excellent option in well-selected cases but this enthusiasm must be tempered by the fact that GCT being a benign lesion the patient is likely to have a normal lifespan and a biological reconstruction may in the long term prove to be a more durable option, especially keeping in mind the socio-economic strata to which most of our patients belong.

Simultaneous developments in the field of radiotherapy have facilitated its use in inoperable lesions or as an adjuvant in lesions in the axial skeleton while reducing the risk of malignant transformation which was a danger with the earlier generation orthovoltage therapy.^2^

A better understanding of the biology of this tumor has revealed that stromal cells, which form the main neoplastic component of this tumor, interact with hematopoietic cells in an autocrine manner to regulate the formation of osteoclast-like giant cells that are ultimately responsible for bone destruction. This autocrine regulation may be disrupted by specific therapeutic agents. Bisphosphonates can induce apoptosis in giant cell tumor culture in a dose-dependent manner. Their topical or systemic use carries promise as a novel adjuvant therapy for giant cell tumor by targeting osteoclast-like giant cells, mononuclear giant cell precursor cells and the autocrine loop of tumor osteoclastogenesis.^3^

In spite of all these advances, confusion does occasionally prevail in the minds of orthopedic surgeons as to the ideal method of management of these tumors. Certain controversies continue to intrigue us: Do adjuvants like phenol or cryotherapy for extension of curettage have any benefit?; Is it necessary to pack the defect with bone graft or cement?; Should a recurrent lesion be curetted again or widely excised?; What is the role of chemotherapy or radiotherapy in the management of these lesions?; Does one contemplate joint salvage or resection, especially in large GCTs?; What is a malignant GCT and how is it managed?

As orthopedic surgeons we can all agree to disagree. Though the value of personal experiences should not be disregarded, in this age of “evidence”-based rather than “eloquence/eminence”-based medicine it would be prudent to base our decisions on scientific facts and the results of well-designed clinical studies. It is only by giving due credence to these that we can aim to do justice to our patients and ensure that they receive the best possible care.
